# Rapid Glomerulotubular Nephritis as an Initial Presentation of a Lethal Diquat Ingestion

**DOI:** 10.1155/2021/4723092

**Published:** 2021-09-11

**Authors:** Daniel Guck, Reynaldo Hernandez, Steven Moore, Andry Van de Louw, Philippe Haouzi

**Affiliations:** ^1^Division of Pulmonary and Critical Care Medicine, Department of Medicine, Pennsylvania State University, College of Medicine, Hershey, PA, USA; ^2^Department of Emergency Medicine, Pennsylvania State University, College of Medicine, Hershey, PA, USA

## Abstract

**Introduction:**

Diquat is an herbicide that can lead to rapid multiorgan system failure upon toxic ingestion. Although Diquat shares a similar chemical structure with paraquat, diquat is still readily available to the general population, and in contrast to paraquat, it is not regulated. We present a case of an intentional diquat poisoning which emphasizes the necessity of the early recognition due to atypical symptoms within the first 24 hours and certainly enhanced regulatory restrictions on this very toxic compound.

**Case:**

A 60-year-old male with a history of severe depression presented to the emergency department after intentional ingestion of a commercial herbicide containing diquat dibromide 2.30%. The earliest manifestations of this acute diquat intoxication comprised a glomerulonephritis and proximal tubular dysfunction. Progressive multiorgan system failure then developed with a significant delay (24–38 hours) including acute renal, liver failure, and then respiratory failure with refractory hypoxemia. Despite maximal supportive care, the end organ failure was lethal. *Discussion*. Diquat intoxication should be suspected in patient presenting an acute glomerulonephritis with coma. Diquat should undergo the same regulatory restrictions as paraquat-containing compounds.

## 1. Introduction

Diquat (1, 1′-ethylene-2, 2′-bipyridylium) is an herbicide used commercially to treat terrestrial and aquatic vegetation [1]. The toxidrome of diquat intoxication is usually considered to be very similar to that of paraquat, since both agents share a similar chemical structure. However, their mechanisms of toxicity remain poorly understood but seem to involve a rapid oxidative stress and superoxide radical production [2]. The resultant clinical toxic manifestations range from mild local irritative effects to multiorgan system failure and death, in keeping with the dose ingested [2]. We present a case of an intentional diquat ingestion leading to early neurological and renal toxicity which developed into a delayed fatal multiorgan failure. This emphasizes important recognizing the timeline of the symptoms of an acute diquat intoxication and the necessity to apply early aggressive interventions to reduce gastrointestinal absorption. Last, the regulatory restrictions currently in place for paraquat should be applied to diquat.

## 2. Case Presentation

**Figure 1:**
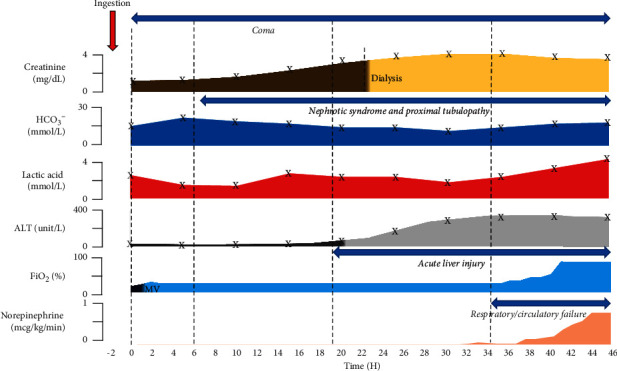
Schematic presentation of the clinical course presented by the patient from admission to the fatal outcome. Trends of the most relevant blood tests, the inspired fraction of O_2_ required to maintained SaO_2_ > 90%, and the level of vasopressor (norepinephrine) support after diquat ingestion are displayed. ALT, alanine aminotransferase; HCO_3_^−^, bicarbonate; MV, mechanical ventilation.

A 60-year-old male with a history of severe depression presented to the emergency department two hours after an intentional ingestion of ∼500 milliliters of a commercial herbicide containing diquat dibromide 2.30%. On initial examination, the patient did not show any hypoxemia, circulatory, or renal failure, but had depressed mental status. Within thirty minutes, a rapid decline in mental status prompted intubation for airway protection. Direct laryngoscopy during the intubation revealed mucus membrane erosion in the posterior pharynx. The orogastric tube was placed after intubation, and 400 ml of green liquid was suctioned from the patient's stomach. Activated charcoal was not administered per recommendation of our poison control center due to the delayed arrival of the patient to the emergency department after ingestion. Blood diquat levels determined within 4 hours following the ingestion reached 9.9 *µ*g/ml (a concentration typically associated with a lethal outcome [3]). After transfer to the medical intensive care unit, the patient was started on a combination of N-acetylcysteine, ascorbic acid, vitamin E, and dexamethasone. Within twenty-four hours following the ingestion, the patient presented a nephrotic range proteinuria (urine protein: creatinine >32 g/day) along with marker of proximal tubular dysfunction including glycosuria, phosphaturia, and increased bicarbonaturia. An acute kidney injury later developed (GFR 17 mL/min/1.73 m^2^) with worsening anion gap metabolic acidosis requiring continuous renal replacement therapy as illustrated in Figure 1. At the same time, a rapid elevation in transaminases and signs of hepatic failure developed (INR 1.4). The respiratory status deteriorated 36 hours only after ingestion with a refractory hypoxemia and imaging of the chest compatible with ARDS. While transthoracic echocardiogram performed and the circulatory status remain within the normal range at 24 hours, an irreversible shock with hyperlactacidemia requiring vasopressor support developed around 36 hours. Despite maximal medical therapy, multiorgan system failure was fatal.

## 3. Discussion

There is limited understanding of the mechanisms of diquat toxicity and no clear rationale for an effective strategy of treatment. Diquat ingestion, in contrast to paraquat, appears to produce very early adverse neurological syndromes (depressed mental status) due to either direct or secondary neurologic injury after ingestion [4]. As diquat is renally excreted, tubular damage results in glomerulonephritis with proximal tubular necrosis developing within 24 hours and resulting in glucosuria and proteinuria, often without diuresis [5]. This is often accompanied by liver injury [6]. Only much later, acute hypoxic respiratory failure and refractory shock can develop. This sequence differs from that produced by paraquat which produces an early pulmonary toxicity and delayed neurological symptoms. Agents counteracting the oxidative stress have been proposed for treating paraquat intoxication [7], but we have currently no standard of care for diquat poisoning. The primary treatment goal is to prevent gastrointestinal absorption either by gastric suctioning and administration of activated charcoal or Fullers earth [8]. More research on the mechanisms of toxicity and treatments of poisoning by this family on compounds is certainly warranted. Finally, it is unclear why diquat is still a readily available herbicide, while paraquat usage is tightly regulated, as both chemicals, despite some differences, are extremely toxic.

## Data Availability

The data used to support the findings of this study are from the electronic medical record, which is unavailable for public viewing.
